# Comparison of *Interferon-Gamma* (IFNG) +874 T/A Single Nucleotide Polymorphism in Hepatitis C Virus Infected Patients and Non-Infected Controls in Mashhad, Iran

**Published:** 2017-05-31

**Authors:** Sina Rostami, Alireza Pasdar, Sina Gerayli, Hamed Hatami, Samaneh Sepahi, Fatemeh Nategh, Mojtaba Meshkat, Seyed Mousalreza Hoseini, Mitra Ahadi, Hamid Reza Sima, Hasan Vosughinia, Mohammad Reza Sarvghad, Abbas Esmaeelzade, Hosein Nomani, Homan Mosanan Mozafari, Fariba Rezai Talab, Mohammad Taghi Shakeri, Zahra Meshkat

**Affiliations:** 1 *Dept. of Laboratory Medicine, Children's and Women's Health, NTNU - Norwegian University of Science and Technology, NO-7491 Trondheim, Norwa​y*; 2 *Dept. of Modern Sciences and Technologies, Faculty of Medicine, Mashhad University of Medical Sciences, Mashhad, Iran*; 3 *Division of Applied Medicine, Medical School, University of Aberdeen, Foresterhill, Aberdeen, AB25 2ZD, UK*; 4 *Dept. of Biology, University of Western Ontario, London, Ontario N6A5BF, Canada*; 5 *Dept. of Immunology, School of Medicine, Mashhad University of Medical Sciences, Mashhad, Iran*; 6 *Targeted Drug Delivery Research Centre, School of Pharmacy, Mashhad University of Medical Sciences, Mashhad, Iran*; 7 *Applied Biotechnology Research Center, Tehran Medical Sciences Branch, Islamic Azad University, Tehran, Iran*; 8 *Islamic Azad University, Mashhad Branch, Mashhad, Iran*; 9 *Dept. of Internal Medicine, Qaem Hospital, Mashhad University of Medical Sciences, Mashhad, Iran*; 10 *Dept. of Internal Medicine, Imam Reza Hospital, Mashhad University of Medical Sciences, Mashhad, Iran*; 11 *Dept. of Infectious Diseases, Imam Reza Hospital, Mashhad University of Medical Sciences, Mashhad, Iran*; 12 *A* *ntimicrobial* * Resistance Research Center, Mashhad University of Medical Sciences, Mashhad, Iran*; 13 *Dept. of Biostatistics, Public Health School, Mashhad University of Medical Sciences, Mashhad, Iran*

**Keywords:** Interferon-gamma, Single Nucleotide Polymorphism, Hepatitis C virus

## Introduction

### Background and Objectives:

Interferon-gamma is an important cytokine, which facilitates immunity against intracellular pathogens. Several factors, including genetic variations of cytokine-producing genes have been shown to influence the progression and severity of Hepatitis C virus (HCV) infection.

### Methods:

Between January and December 2012, 87 HCV-infected individuals and 89 individuals without HCV infection were recruited for the study of Single Nucleotide Polymorphism (SNP) at Interferon Gamma *(IFNG)* +874 T/A. After extraction of genomic DNA from Peripheral Blood Mononuclear Cells (PBMCs) in blood sample of the individuals, Amplification Refractory Mutation System (ARMS) polymerase chain reaction was performed to evaluate the SNP at this position.

### Results:

 The frequency of genotype TA was 62.1% in the HCV-infected group, while it was 47.2% for the control group (p=0.033). However, after adjusting for confounders (including alcohol consumption, drug addiction, transfusion, and tattoos), the genotypes at this position did not show any statistically significant association with HCV infection (adjusted P values were above 0.05). The frequency of allele A was slightly higher in patients than the controls (55.2% versus 48.3%).Carriers of A allele were more frequent in patients with HCV infection compared to the control group (55.17% in patients versus 48.31% in the control group; P=0.02). However, after adjustment for confounders, the results were no longer statistically significant (P=0.2).

### Conclusion:

A carrier status for certain alleles and genotypes at Interferon Gamma *(IFNG)* +874 T/A may lead to higher susceptibility to HCV infection in a certain population.

## Introduction

Chronic infection with Hepatitis C Virus (HCV) may lead to complications such as fibrosis, cirrhosis, and Hepatocellular Carcinoma (HCC). Half a million new cases of liver cancer have been estimated, annually, 22% of which are caused by infection with HCV (1). Worldwide, about 130 to 170 million individuals are infected with Hepatitis C Virus (HCV) (2).

Attempts to develop therapeutic vaccines against HCV infection have not so far been very successful as a result of variants of HCV, which are capable of undergoing extensive mutations that confer them new resistance adaptations (3). An area that has received extensive attention during the recent decades is the study of host immunity and its interactions with infectious agents, rather than the previously more conventional approach of studying variations in infectious agents. It has been shown that chronic HCV effects the number, subset composition, and functional performance of immune cells, including natural killer cells, natural killer T cells, dendritic cells, macrophages, and T cell (4). Furthermore, genetic variations in host genes, which have a role in mediating immunity, may affect the regulation and production of components required for the two arms of the immune system, namely innate and adaptive immunity. One of the important components is cytokines, which have immune regulatory roles and are secreted by the immune cells. Cytokines are capable of suppressing viral replication and mediating host immune responses (5). Cytokines may show different expression levels, while interactions with other components may affect the ability of the immune system to combat the infection and to reduce or eliminate the virus or its associated complications. An estimated 10 million Single Nucleotide Polymorphisms (SNPs) are assumed to be present in the human genome (6).It has been suggested that the rate of expression of some cytokines is influenced by mutations in their regulatory and coding regions. This, in turn, is considered to influence the natural history of HCV infection (7, 8). 

One of the important cytokines involved in suppressing viral infections is Interferon Gamma (*IFNG*), which has clinical implications in preventing the development of chronic hepatitis, liver cirrhosis, and hepatocellular carcinoma (9). The *IFNG* gene is present on chromosome12q24.1. Interferon Gamma-induced signaling pathways involved in the activation of genes with antiviral properties are very complicated (10). The signaling pathway is partly controlled by the Ras-MAPK and Jak-STAT pathways (11, 12). Antiviral properties of *IFNG* are mediated through the induction of gene expression for double-stranded RNA activated Protein Kinase (PKR), 2’-5’ oligoadenylate synthetase, and dsRNA-specific adenosine deaminase (13). In addition to its role as an antiviral agent, IFNG is capable of engaging T cells to the site of inflammation (14, 15). The IFNG produced by T and Natural Killer (NK) cells can mediate the function of T helper cell type 1 (Th1) (16)

The SNP at Interferon Gamma (*IFNG*) +874 T/A of the human Interferon-Gammais present at the 5' end of the first intron. Polymorphisms normally appear in regulatory regions and their role in certain diseases has been previously investigated. Among these polymorphisms, intron 1 +874 T/A and C/A repeat microsatellites and promoter SNP -179 T/G have been considered previously (17). Although some in vitro studies showed that IFNG can suppress the replication of HCV (18, 19), no correlation has been found regarding the expression level of IFN Gand HCV viral load (20, 21).

The current study was performed to evaluate the frequency of alleles and genotypes at position +874 of the *IFNG* gene between patients infected with HCV and individuals without HCV infection in the city of Mashhad, northeast of Iran, to determine if there is any association between a specific allele or genotype at this position and infection with chronic HCV.

## Materials and Methods

**Population study and sample collection**

After receiving the approval from the ethical committee of Mashhad University of Medical Sciences, 87 HCV-infected patients and 89 healthy individuals were recruited from January to December 2012 for the study of single nucleotide polymorphism at* IFNG *+874T/A. Informed consents were obtained from the studied patient and controls. Blood samples were obtained at the Microbiology and Virology Research Center of Qaem hospital and outpatient clinic of Qaem hospital in Mashhad, Iran. Ten- and three-milliliter blood samples were obtained from patient and control groups, respectively. Blood samples were collected in tubes containing EDTA. Genomic DNA (for Amplification Refractory Mutation System (ARMS) Polymerase Chain Reaction (PCR)) was extracted from whole blood using a genomic DNA extraction Kit (GeNet Bio, Daejeon, Korea). Viral RNA was isolated from sera using QIAamp Viral RNA Mini Kit (Qiagen, USA). The control group was defined as individuals without HCV infection as determined by commercial Enzyme Linked Immunosorbent Assay (ELISA) kits (Delaware Biotech, USA). Control subjects were screened for HBV and HCV markers by HBsAg and anti-HCV antibody ELISA kits (Delaware Biotech, USA). Hepatitis C Virus-infected patients were evaluated based on positive anti-HCV IgG test results as determined by a commercial ELISA kit (Delaware Biotech, USA), and HCV RNA, using Reverse Transcriptase (RT)-PCR, as described previously (22). Hepatitis C Virus genotyping was performed using the method described by Ohno et al*. *(23). The HCV RNA load was evaluated using Gene Proof HCV real-time PCR Kit (Gene Proof a.s., Czech Republic) in accordance with the manufacturer’s instructions. Three standard concentrations from 10^2^ to 10^4^ copy/mL were made, and the real-time PCR was performed on Rotor Gene (Corbett Research, Australia). All known factors involved in the risk of HCV transmission, including yet not limited to a history of drug and alcohol addiction, transfusion, HIV infection, tattoos, working environments such as working in hospitals and family history of HCV infection, were recorded for the studied sample. A summary of demographic and risk factors are given in Table 1.

**Table 1:** Information on demographic and risk factors in the studied population of HCV patients and control groups

Characteristics	Patients	Controls	P-Value
Male: Female	78:9	39:50	<0.05
Age ( years; mean ± SD )	42.58+10.28	36.33+12.72	<0.05
Addiction	37 (42.5%)	-	<0.05
Alcohol	34 (39.1%)	2 (2.2%)	<0.05
Transfusion	31 (35.6%)	7 (7.8%)	<0.05
Tattoo	27 (31.0%)	-	<0.05
ALT (U/L, mean ± SD)	36.42+30.59	27+21.45	0.06
Increased ALT*	26 (29.9%)	19 (21.35%)	<0.05

* ALT, alanine aminotransferase with the unit U/L (unit per liter). * >41 U/L for men and >31 U/L for women.

**DNA extraction and genotyping**

Following extraction of DNA, ARMS PCR was performed. The primer sequences used for the amplification of IFNG+874T/A (238 bp in length) were as follows: T allele-specific primer, 5'-TTCTTACAACACAAAATCAAATCT-3'; A allele-specific primer, 5'–TTCTTACAACACAAAATCAAATCA3' and reverse primer 5'-TCAACAAAGCTGATACTCCA3'. For PCR, the final reaction volume was 20 µL including: 1X PCR buffer, 10 pmol forward and reverse primers, 10mM MgCl_2_, 1.5 mMdNTPs, and 0.5 unit Taq DNA polymerase (GeNetBio, Korea). The PCR program included initial denaturation at 96°C for 5 minutes and one cycle of 96°C for 130 seconds, 63°C for 60 seconds, and 72°C for 30 seconds; 9 cycles of 96°C for 10 seconds, 63°C for 60 seconds, and 72°C for 30 seconds, followed by 20 cycles of 96°C for 10 seconds, 60°C for 50 seconds and 72°C for 30 seconds. The PCR was followed by a final extension at 72°C for 5 minutes. Amplified PCR products were analyzed by 2.5% agarose gel stained with Green Viewer (Pars Tous, Iran). 

**Biochemical analysis**

Alanine aminotransferase levels were determined for the studied sample using Pars Azmoon Kit (Pars Azmoon, Iran) in accordance with the manufacturers’ recommendations. A summary of the results of ALT is presented in Table 1. Alanine aminotransferase levels (determined at 37°C) of < 31 U/L for females and < 41 U/L for males were reported as normal.

**Statistical analysis**

Chi-square and Fisher’s exact test were used for qualitative comparison. Qualitative comparison was performed with t test. Logistic regression was used to compare the two groups. Data analysis was carried out using IBM SPSS software (IBM Corp., Armonk, NY) version 21 (24). P values below 0.05 were considered statistically significant.

## Results

**Population study**

**Figure 1:**
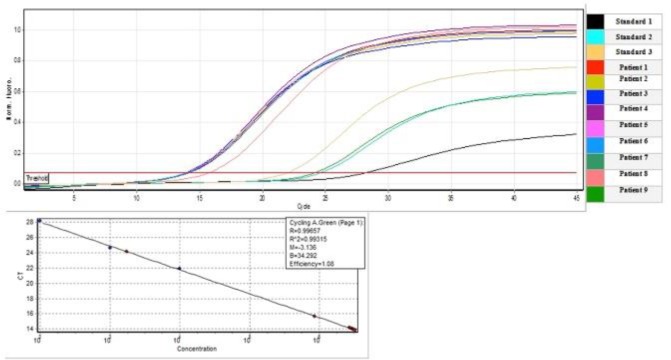
Viral Load Results of Nine Patient Samples and Three Standards

The mean age of the patients was 42.58± 10.28 years while that of the control group was 36.33± 12.72 years. The age ranged from 16 to 66 years and 15 to 65 years in the patient and control groups, respectively. Also, among the patient group, 78 (89.7%) were male and 9 (10.3%) were female. While in the control group 39 (43.8%) were male and 50 (56.2%) were female (Table 1). The duration of treatment with interferon and ribavirin varied from one month to two years in patients with chronic HCV infection. The authors were not able to recruit more females in the patient group firstly because the above-mentioned high-risk behaviors were more commonly observed among males in the studied population and secondly because a lower number of females were willing to take part in the present study. Real-time PCR was used to evaluate HCV load for all participants in the patient group. The viral load results for nine patients are shown in Figure 1. 

**Genetics study**

**Figure 2:**
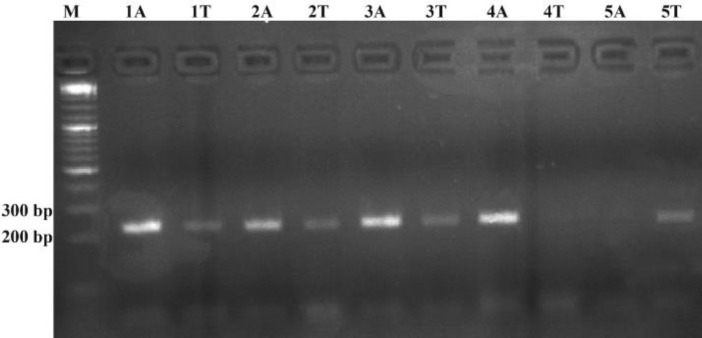
Agarose Gel Result of the Polymerase Chain Reaction Products of IFNG Gene +874 T/A Polymorphism

The agarose gel results of ARMS-PCR for several polymorphism statuses at IFNG +874 T/A is shown in Figure 2. 

Viral load analysis was performed by the Real-time PCR method using a commercial kit (R=0.996, efficiency=1.08).

Genotype TA: sample 1 (lanes 1A and 1T), sample 2 (lanes 2A and 2T), and sample 3 (lanes 3A and 3T); genotype AA: sample 4 (lanes 4A and 4T); genotype TT: sample 1(lanes 5A and 5T), lane M: 100 bp DNA size marker

The current study suggested that the *IFNG *+874T/A gene polymorphism had different genotype and allele frequencies between patient and control groups. All genotypes in cases and controls were in Hardy-Weinberg Equilibrium. The frequency of the TA genotype was 62.1% in the patients, while it was 47.2% for the control group (p=0.033). The frequency of genotype AA was almost the same in both groups (24.7% and 24.1% among control and patient groups, respectively). However, after adjusting for confounders (including alcohol consumption, drug addiction, transfusion, and tattoos), the genotypes at this position did not show any statistically significant association with HCV infection (adjusted P values were above 0.05). The frequency of the A allele was slightly higher in patients than the controls (55.2% versus 48.3%), while the frequency of the T allele was higher in controls than patients (51.7% versus 44.8%) (Table 2). 

Based on a dominant model, carriers of the A allele were more frequent in patients with HCV infection compared to the control group (55.17% in patients versus 48.31% in the controls; P=0.02). However, after adjustment for confounders, the results did not remain statistically significant (P=0.2) (Table 3).

**Table 2:** Distribution of genotypes and allele frequencies of Interferon-gamma (+874) T/A single nucleotide polymorphism among control and patients groups.

IFN-Gamma locus	Control (n = 89)	Frequency (%)	Patients (n = 87 )	Frequency (%)	P value	OR		95% C.I.
Genotype Frequencies								
TT	25	28.08	12	13.79	0.02	0.41		(0.19-0.87)
AA/AT	64	71.90	75	86.21				
Allele Frequencies								
A	86	48.31	98	55.17	0.02	1.31	(0.86 1.98)	
T	92	51.69	80	44.83				

**Table 3:** Adjusted^*^ values for genotypes and allele frequencies of Interferon-gamma (+874) T/A single nucleotide polymorphism among control and patients groups.

IFN-Gamma locus	Control (n = 89)	Frequency (%)	Patients (n = 87 )	Frequency (%)	P value	OR	95% C.I.
Genotype Frequencies							
AA	22	24.72	21	24.14	Ref		
AT	42	47.19	54	62.07	0.42	0.74	0.36-1.53
TT	25	28.09	12	13.79	0.14	1.99	0.80-4.95
AA	22	24.71	21	24.13	0.93	0.97	(0.49-1.93)
TT/AT	67	75.27	66	75.85			
TT	25	28.08	12	13.79	0.4	0.60	(0.20-1.83)
AA/AT	64	71.90	75	86.19			
Allele Frequencies							
A	86	48.31	98	55.17	0.20	1.31	(0.86-1.98)
T	92	51.68	80	44.82			

* Adjusted for alcohol consumption, drug addiction, transfusion and tattoos

There was no significant association between HCV genotypes and *IFNG* genotypes in patients (P>0.05). The study did not find any significant association between HCV RNA titer and either the HCV genotypes or *IFNG* genotypes (P>0.05). 

**Biochemical assay**

**Figure 3:**
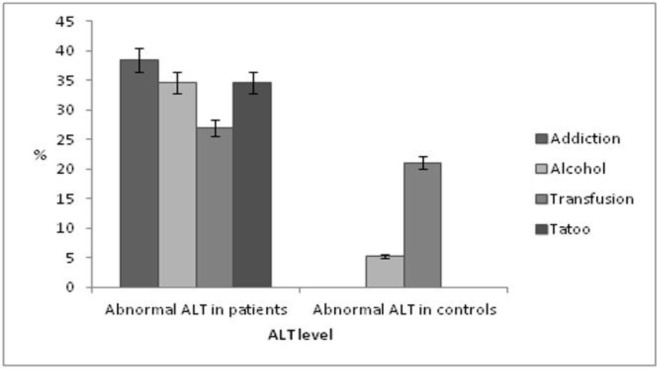
The Relationship between Alanine Aminotransferase Levels in the Controls and Patients and Common Risk Factors of Hepatitis C Virus Infection. ALT: alanine aminotransferase

Biochemical results showed that mean ± Standard Deviation (SD) of ALT levels in patients and controls were 36.42+30.59 and 27.00+21.45, respectively (P=0.06). The relationship between ALT levels (evaluated based on stratification in normal and abnormal ALT level groups) in the control and patient groups and common risk factors of HCV infection are shown in Figure 3.

## Discussion

The prevalence of HCV was shown to be below 1% in the general population of Mashhad, northeast of Iran, with genotypes 1a (41.7%) and 3a (33.0%) being the most prevalent genotypes in this region (24, 25). As expected, addiction, alcohol consumption, transfusion, and tattoos had a statistically significant association (p<0.05) with HCV infection in the studied population. Although higher ALT levels were observed in the patient group (ALT (U/L), mean ± SD=36.42+30), compared to the control group (ALT (U/L, mean ± SD=27+21.45), a statistically significant difference was not observed for the ALT levels (p-value = 0.06). The result of genotype analysis of a dominant analysis model of the IFNG +874 T/A position (TT vs. AA+AT) indicated that the mixed AA+AT genotype was more common in patients (p=0.02; OR=0.41, 95% CI: 0.19 - 0.87).

Viral genetic factors and variations, including different genotypes and quasi species of HCV, and host factors including, gender, age, duration of infection, and history of alcohol intake or diabetes could influence the outcome of HCV infection (26, 27). Ethnicity has been shown to influence the inheritance of specific patterns of polymorphisms in cytokine genes (28). Polymorphisms in genes related to both adaptive and innate immunity are considered to be crucial in clearance of HCV infection (29). 

Replication of HCV genome and the severity of the disease are known to be influenced by the expression of the *IFNG *gene (30). Phenotypically, individuals having T/T, T/A, and A/A genotypes at this position are high, intermediate, and low producers, respectively (31). 

In our study, both genotype and allele frequencies at *IFNG* +874 T/A showed a statistically significant association with HCV infection (p<0.05). However, after adjustment for confounders (alcohol consumption, drug addiction, transfusion, and tattoos) the results did not remain statistically significant for both allele and genotype frequencies (p>005). This may indicate that the observed trend in the association of some alleles and genotypes with HCV infection was not entirely dependent on the polymorphism at this position and that the confounders, which are normally associated with HCV infection had more profound effects. 

The association of *IFNG* +874 T/A with several other infectious diseases has been studied so far. In a study from Brazil on patients with ocular toxoplasmosis and the control group (without the infection), similar to our study, after adjustment for confounders, no statistically significant difference was observed for A and T alleles (p-value=0.113 ;). In that study, however, unlike our study, which indicated an almost similar genotype frequency for AA genotype for both groups of HCV-infected patients and the control group (about 24% genotype frequency of AA in both groups), the AA genotype was more frequent in the patient group with ocular toxoplasmosis when compared with the control group (44% of patients vs. 23% of controls had AA) (32). With regards to other infectious diseases, a study in Turkey suggested that the TT genotype had a higher frequency in patients with brucellosis (33). In other studies, the A allele of *IFNG* +874 had also been shown to influence the outcome of diseases such as parvovirus and tuberculosis (34, 35). 

Several studies have also noted the importance of polymorphism at *IFNG* +874 T/A with Hepatitis B Virus (HBV) infection. Among them, a study among Korean patients with HBV infection did not provide evidence in favor of the association between polymorphism at *IFNG* +874 T/A and persistent HBV infection (36). This finding was also confirmed in a study on a Turkish population, in which no association was found between *IFNG *+874 T/A polymorphism and HBV persistence (37). A study sample of patients from North China, however, showed the association of AA genotypes with persistent infection with HBV, HCV, and HBV/HCV co-infection (38).

With regards to HCV infection, several studies provided evidence for the significance of this polymorphism on response to treatment and severity of the disease. A study from Taiwan on chronic HCV infected patients did not suggest any association between the inheritance of A or T allele at *IFNG* +874 and response to combination therapy with high-dose Interferon (IFN)-alpha and ribavirin (39). A similar result was also observed among Iranian patients with HCV infection, who were treated with Pegylated (PEG)-IFN plus ribavirin. In that study, no statistically significant difference was observed between allele and genotype frequencies between the responder and non-responder patients (40). The inheritance of the T allele at this position was considered to be associated with high rate of cirrhosis compared to carriers of homozygote A allele (p-value: 0.028) (41). Polymorphism of *IFNG *was shown to have a role in recurrent hepatitis C after transplantation. It was shown in a study that 80% of non-recurrent patients had the polymorphism associated with higher levels of IFNG production (the T/T genotype) (42).

A study among Tunisian patients with HCV infection and a control group, similar to our study, indicated no statistically significant difference in allele or genotype frequencies between the patient and control groups at *IFNG* +874(43).

Other SNPs have been reported in IFNG gene polymorphisms that may have more profound significance, for instance, the -756 G SNP in the proximal *IFNG* promoter region was found to have more functional importance in HCV persistence (44), and two SNPs in *IFNG* R2 were found to be associated with HBV viremia. Similarly, SNPs within *IFN-AR2 *were reported to have associations with persistent HBV infection in Gambia (45).

## Conclusion

Taking into account the result of several studies, it seems that ethnicity is partly involved in susceptibility to diseases including hepatitis C virus, in which genetic polymorphisms may confer different levels of risk in different populations. Immunity is complex and many pathways, including signaling pathways are affected by the interaction of environmental and genetic factors. The difference in polymorphism of one cytokine does not directly influence the severity and the progression of a disease; it is rather the collective profile of cytokine genes, components of signaling pathways and other related genes, which determine the outcome. Even in a certain population, more accurate observations require a huge sample size representing patients with full history of therapies they have undergone, risk factors, and relevant data over a period of time. For future studies, it is preferable to use high-throughput methods of genotyping to take into account the combination of a larger number of polymorphisms in immune-related genes and also to recruit a larger sample size of patients with a complete set of data.
